# Impact of Spinal Manipulation on Cortical Drive to Upper and Lower Limb Muscles

**DOI:** 10.3390/brainsci7010002

**Published:** 2016-12-23

**Authors:** Heidi Haavik, Imran Khan Niazi, Mads Jochumsen, Diane Sherwin, Stanley Flavel, Kemal S. Türker

**Affiliations:** 1 Centre for Chiropractic Research, New Zealand College of Chiropractic, Auckland 1060, New Zealand; imran.niazi@nzchiro.co.nz (I.K.N.); tadarida7@gmail.com (S.F.); 2 Centre for Sensory-Motor Interaction, Department of Health Science and Technology, Aalborg University, Aalborg 9220, Denmark; mj@hst.aau.dk; 3 Chirofit (Private Practice), 32a Normanby Rd, Mt Eden Auckland, Auckland 1024, New Zealand; diane@chirofit.co.nz; 4 School of Medicine, Koç University, Rumelifeneri Yolu, Sariyer, Istanbul 34450, Turkey; kturker@ku.edu.tr

**Keywords:** transcranial magnetic stimulation, movement related cortical potential, neural adaptations

## Abstract

This study investigates whether spinal manipulation leads to changes in motor control by measuring the recruitment pattern of motor units in both an upper and lower limb muscle and to see whether such changes may at least in part occur at the cortical level by recording movement related cortical potential (MRCP) amplitudes. In experiment one, transcranial magnetic stimulation input–output (TMS I/O) curves for an upper limb muscle (abductor pollicus brevis; APB) were recorded, along with F waves before and after either spinal manipulation or a control intervention for the same subjects on two different days. During two separate days, lower limb TMS I/O curves and MRCPs were recorded from tibialis anterior muscle (TA) pre and post spinal manipulation. Dependent measures were compared with repeated measures analysis of variance, with *p* set at 0.05. Spinal manipulation resulted in a 54.5% ± 93.1% increase in maximum motor evoked potential (MEPmax) for APB and a 44.6% ± 69.6% increase in MEPmax for TA. For the MRCP data following spinal manipulation there were significant difference for amplitude of early bereitschafts-potential (EBP), late bereitschafts potential (LBP) and also for peak negativity (PN). The results of this study show that spinal manipulation leads to changes in cortical excitability, as measured by significantly larger MEPmax for TMS induced input–output curves for both an upper and lower limb muscle, and with larger amplitudes of MRCP component post manipulation. No changes in spinal measures (i.e., F wave amplitudes or persistence) were observed, and no changes were shown following the control condition. These results are consistent with previous findings that have suggested increases in strength following spinal manipulation were due to descending cortical drive and could not be explained by changes at the level of the spinal cord. Spinal manipulation may therefore be indicated for the patients who have lost tonus of their muscle and/or are recovering from muscle degrading dysfunctions such as stroke or orthopaedic operations and/or may also be of interest to sports performers. These findings should be followed up in the relevant populations.

## 1. Introduction

Over the past 15 years there has been a growing number of human experiments conducted that have demonstrated there are central neural plastic effects from manual spinal manipulation [1]. Spinal manipulation is a conservative, low cost treatment option currently utilized for low back pain, neck pain and headaches. Clinical trials and systematic reviews have shown its usefulness for these conditions [2,3,4,5]. However, the mechanism that underpins the functional recovery and amelioration of painful conditions remains poorly understood. Scientists use to believe spinal manipulation was a biomechanical treatment option for spinal pain conditions. However, the growing basic science evidence suggests there may be more of a neurophysiological effect following spinal manipulation than previously realized.

Several of these basic science studies have suggested that the central plastic changes observed following manipulation occur at the cortical level [6,7,8,9,10,11,12]. Spinal manipulation of dysfunctional joints has been shown to attenuate cortical (parietal N20 and frontal N30) somatosensory evoked potential (SEP) responses [7]. In the original study, the frontal N30 changes persisted on average for 20 min post-manipulation before returning to baseline levels, whereas the parietal N20 changes persisted for at least 30 min, i.e., during all three post-manipulation recordings [7]. In several additional studies the changes in N30 SEP peak were confirmed [10,11]. Most evidence suggest this SEP peak has multiple neural generators and reflects processing within a complex cortical and subcortical loop linking the post-central cortical regions (i.e., S1) [13,14,15], the basal ganglia, thalamus, pre-motor areas, and primary motor cortex [16,17,18,19,20]. The frontal N30 peak is therefore thought to reflect early sensorimotor integration [21]. Recently, a study using whole head electroencephalography (EEG) and brain electrical source analysis was able to further explore which brain sources are responsible for these changes in N30 amplitude following a single session of spinal manipulation [22]. Using dipole source localization it was demonstrated that this change in sensorimotor integration that occurs after spinal manipulation predominantly happens in the prefrontal cortex [22]. 

Additional studies have explored whether these SEP change following spinal manipulation of dysfunctional joints might reflect improved proprioceptive processing. One study was conducted to investigate whether cervical manipulation improved elbow joint position sense [12]. This study did show that manipulating the neck of the participants who had a history of neck dysfunction, but who were not in pain on the day of the experiment, did improve their elbow joint position sense [12]. Another study has shown that chiropractic care for 12 weeks improved ankle joint position sense in older adults [23]. 

Motor control changes following spinal manipulation have also been demonstrated. One study has shown that spinal manipulation of dysfunctional neck joints altered cortical motor control of two upper limb muscles in a muscle-specific manner, using transcranial magnetic stimulation (TMS) of the brain [9]. This study used a paired-pulse TMS protocol to explore specific central corticomotor facilitatory and inhibitory neural pathways to two target muscles. Another study has shown that spinal manipulation improves tibialis anterior (TA) strength, and that this change most likely comes from the supraspinal regions as only very small but significant changes in the H-reflex was observed at low intensities while large changes were shown in the V wave [24]. 

Taken together, these findings suggest that spinal function and/or movement has a significant impact on central cortical processing that improves the accuracy with which the brain is aware of limb position and alters the way the brain controls upper and lower limb movement patterns. If manual spinal manipulation does result in an increase in cortical excitability, this could have far-reaching consequences for a variety of patient populations, where numerous invasive neuromodulatory treatment modalities are currently being trialled with varying degrees of success. These treatment modalities include techniques such as transcranial direct current stimulation (tDCS) and repetitive transcranial magnetic stimulation (rTMS) [25,26,27]. As spinal manipulation is relatively accessible, inexpensive and safe it could be a conservative treatment worth trialling before more expensive and invasive options are considered, not only for pain syndromes, but also for a variety of conditions that spinal manipulation has not previously been considered for. 

Although the previous TMS study did not show changes in single pulse, TMS evoked motor evoked potential (MEP) amplitudes in the upper limb muscle abductor pollicis brevis (APB) at mid-range brain stimulation intensities, the paired-pulse TMS findings for the APB showed decreased specific intracortical inhibitory pathways alongside increases in intracortical facilitatory pathways to the same muscle [9]. We therefore hypothesized that looking at stimulus response curves of this upper limb muscle may demonstrate increases in single pulse MEPs at higher or lower stimulus intensities, as this has not previously been investigated. In addition, considering the significant improvements in TA strength (maximum voluntary force) likely originated from the cortex following spinal manipulation [24] we also hypothesized we would find a shift of the TMS stimulus response curve for TA, at least at higher intensities. Finally, as most of the previous studies show changes in cortical or intracortical processing and integration we hypothesized that we would be able to observe changes in the movement related cortical potential (MRCP), as it is known to reflect cortical changes that occur prior to any actual movement initiation [28].

This study therefore sought to investigate whether: (1) increases in central excitability are possible following cervical/full spinal manipulation; and (2) whether these changes occur at the cortical level.

## 2. Materials and Methods

The study group consisted of 28 subclinical pain subjects (17 male and 11 female, total mean age 27.6 ± 4.3 years old). Exclusion criteria consisted of current pain on the day of the experiment, a history of having previously sought treatment for spinal pain, a history of neurologic disease or any known contraindications to either spinal manipulation (such as a recent history of trauma, known conditions such as inflammatory or infectious arthropathies, or bone malignancies) or TMS as suggested in [29]. To be included, subjects needed to have a history of mild intermittent spinal pain, ache or tension (subclinical spinal pain), and evidence of dysfunction in the spinal and/or pelvic joints. Evidence of dysfunction included tenderness to palpation of the relevant joints, palpable restriction of intersegmental range of motion, palpable asymmetric intervertebral muscle tension, and any abnormal or blocked joint play and end-feel of the joints. These inclusion and exclusion criteria ensured that all the participants fit the criteria of sub-clinical spinal pain [30,31,32]. Subclinical pain (SCP) refers to recurrent spinal ache, pain or stiffness for which the person has not yet sought treatment. Most of the participants were novice to chiropractic. The Northern Y Regional Ethics Committee approved this study (ref: NTY/07/05/054) in accordance with the Declaration of Helsinki and participants gave written consent for experiments.

### 2.1. Experimental Protocol

Three separate experiments were performed to assess the effect of the cervical vs. full spinal manipulation on the corticospinal excitability, and to explore the possible origin of any observed effects (i.e., cortical or spinal changes). Transcranial magnetic stimulation (TMS) was used to assess the central excitability of a target muscle, prior to (pre) and following (post) any intervention. The first experiment consisted of two sessions (*N* = 12) with one cervical spine manipulation intervention session and one control sessions. The control intervention consisted of a passive head movement (PHM). During the intervention session, the effects of cervical spinal manipulation on upper limb central excitability were assessed by recording TMS stimulus response curves. In addition, to assess contribution of spinal pathways F-wave amplitudes and persistence were recorded alongside the stimulus response data before and after both the spinal manipulation and control interventions. The second experiment (*N* = 14) assessed the effects of full spine manipulation on central excitability of a lower limb TA muscle. The third experiment (*N* = 12) explored the contribution of changes at the cortical level following full spine manipulation by investigating the morphology of cortical signals known as movement related cortical potentials (MRCP). This experiment was carried out to explore whether there would be any detectable cortical changes following spinal manipulation as the early phases of the MRCP is known to be cortical in origin. Generators of these cortical signals are well known in the literature, thus if we find changes in morphology of these signals following spinal manipulation, this would suggest spinal manipulation does alter cortical excitability. There was a gap of 6 months between Experiments 1 and 2, whereas Experiment 3 data were collected alongside Experiment 2. Participants were invited to take part in one or more experimental sessions. Ten subjects took part in both Experiments 2 and 3, which were done on separate days.

### 2.2. EEG and EMG Acquisition and Analysis

EEG signals (0.1–300 Hz) were recorded by Grass Model 12 Neurodataacquisition system amplifier and acquired by a CED Power1401 MK2 data acquisition board through spike2 (version 6) software (Cambridge Electronic Design Ltd. (CED), Cambridge, England) sampling at 1024 Hz. Ag/AgCl scalp electrodes were placed according to the International 10–20 system in the positions FP1, FCz, C1, C2, CPz and Cz. Right ear lobes were used as a reference and the ground electrode was placed at the nasion. Surface electrodes (20 mm Blue Sensor Ag/AgCl, AMBU A/S, Denmark) were used to record the electromyographic (EMG) activity of the upper limb (abductor pollicisbrevis (APB)) and lower limb (tibialis anterior (TA)) muscles of the dominant side for all aspects of the experiments. For standardization electrode positioning was marked according to 10–20 system for EEG and for EMG the electrode positions were marked with a permanent marker to enable consistent placement between days. All EMG data were sampled at a frequency of 2 kHz.

### 2.3. TMS

TMS was used to assess the changes in central excitability as it has been shown in the literature to be a reliable method [33,34,35]. A monophasic TMS stimulator (Magstim 200, Magstim Company, Carmarthenshire, UK) with a focal figure of eight double coil (110 and 70 mm diameter) were used to apply single TMS pulses (inducing a posterior to anterior directed current in the brain) to elicit a MEP in the muscle of primary interest (i.e., the TA and APB muscles). The position was marked on subject’s head which ensured that the TMS stimuli were consistently delivered over the same area of the motor cortex during one experimental session. Subsequently, the resting threshold (RTh) was identified. The RTh was defined as the highest stimulus intensity that in no more than 5 of 10 consecutive stimuli evokes an MEP with amplitude of ~50 µV while the muscle is at rest. Following this, 10 MEPs were elicited in the resting target muscle at five TMS intensities: 90%, 100%, 110%, 120%, and 130% of RTh. The stimuli were delivered randomly every 5–7 s. The mean peak-to-peak MEP amplitudes were measured pre and post intervention for each subject. Three variables were extracted from the fitted data: the maximum value (MEPmax), the slope of the steepest part of the curve (k) and the stimulus intensity required to obtain a response that is 50% of the maximum (S_50_).

### 2.4. F Waves

To assess the contribution of spinal pathways during experiment1, F waves were recorded from the subjects’ relaxed APB. The median nerve was stimulated at the wrist with a supramaximal (1.25 Mmax, i.e., 25% above the stimulus intensity that elicited a maximal M wave) electrical stimulation consisting of square-wave constant current pulses with pulse width duration of 0.2 milliseconds, as recommended in the literature [36,37]. Twenty trials were recorded for each subject before and after each intervention (cervical spine manipulation and control). The F wave amplitudes were recorded and expressed as a percentage of the M wave amplitude. The F wave persistence was expressed as a percentage of F waves present in the 20 trials recorded, which are also required for reliable measure of F wave [38].

### 2.5. MRCP

To assess the cortical contribution during Experiment 3 movement related cortical potential (MRCP) were recorded. The subjects were instructed to perform 50 ballistic isometric dorsiflexion contractions of TA at random time intervals, i.e., no external cues were presented for task execution. Subjects had to perform the movement whenever they wanted to at comfortable contraction levels (~20%–30% of maximum voluntary contraction; MVC) to avoid the prospect of fatigue. This paradigm lead to a fully self-paced set of executed movements, although subjects were told to leave at least an interval of roughly five seconds between two consecutive trials. Self-pace MRCP were recorded before and after the full spinal manipulation intervention, to assess the changes in morphology of cortical signals.

#### 2.5.1. Signal Processing

The EEG signals were band pass filtered from 0.05 to 10 Hz, and then down-sampled to 20 Hz. Laplacian spatial filter (LSF) was applied for obtaining a surrogate channel (a linear combination of 5 EEG channels) containing different spatial components from the recorded brain regions. LSF also helped in increasing the signal to noise ratio (SNR). After acquiring the surrogate channel, the EEG signals were divided into epochs, each epoch consisted of 5 s prior and 1.5 s (in total 6.5 s) after onset of movement. The first 3 s of the epoch was considered as noise and the remaining 3.5 s was considered as the signal. The reference for movement onset was estimated as the time instant when the rectified EMG signal amplitude crossed a threshold equal to one tenth of its maximum during an execution of movement. Epochs contaminated with high EOG activity (125 µV) were rejected from further analysis.

#### 2.5.2. Morphology Extraction (MRCP)

Parameters used for morphology determination of MRCP were latencies and amplitude of early bereitschafts-potentials (EBP), late bereitschafts potentials (LBP), and peak negativity (PN). Apart from different phases of the MRCP, the rebound rate of reafferent potential (RAP) of the MRCP was calculated, as this also served as a control parameter since rebound rate is dependent on different force level of associated movement [39,40]. Apart from rebound rate, EMG was used to control for the force level across trials in pre and post spinal manipulation sessions. If the force level was one standard deviation away from the mean force (calculated from EMG during signal epochs in Pre spinal manipulation session) in any trial in pre or post session, it was rejected.

The PN was defined as a local minimum of the averaged signal epoch in surrogate channel. Three first order polynomials were fitted to the distinct phases of the MRCP; i.e., the EBP, LBP and RAP. The onset of EBP was defined as the intersection of the baseline (mean, calculated from 3 s noise epoch) with a first order polynomial fitted to the EBP. Another first order polynomial was fitted to the LBP and the intersection with the fit for the EBP was defined as the onset of the LBP [28]. The rebound rate was defined as the slope of a fitted first order polynomial to the RAP [28]. 

The amplitudes of the EBP were quantified by calculating the mean amplitude between the onset of EBP and LBP. Similarly, the LBP amplitudes were quantified by calculating the mean amplitude between the onset of the LBP and the PN. The PN amplitude was defined as the mean amplitude from the onset of EBP till PN. 

### 2.6. Interventions

The spinal manipulation was performed by a registered chiropractor with 12 years of clinical experience from the staff at the New Zealand College of Chiropractic. A detailed case history was obtained before the intervention was carried out. 

#### 2.6.1. Spinal Manipulation Intervention

This intervention consisted of high-velocity, low-amplitude manipulation of the subjects’ dysfunctional spinal and/or pelvic joints, which were determined by the chiropractor. This spinal dysfunction was “quantified” to some degree prior to and after each spinal manipulation intervention by assessing for tenderness to palpation of the relevant joints, manually palpating for restricted intersegmental range of motion, assessing for palpable asymmetric intervertebral muscle tension, and any abnormal or blocked joint play and end-feel of the joints. All of these biomechanical characteristics are known clinical indicators of spinal dysfunction [41,42]. These findings were documented pre and post each spinal manipulation intervention. The improvements in segmental function following spinal manipulation were also recorded for each subject.

For the cervical and thoracic spine functional assessment the chiropractor would gently move the subjects head and/or upper body passively from the neutral position to the maximal range of lateral flexion in the coronal plane, while palpating over each segment and applying gentle pressure to both the left and the right sides. If this movement appeared restricted, the examiner would apply additional gentle pressure to the joint, while watching for signs of discomfort from the subject. The examiner would also ask the subject if the pressure to the joint elicited pain. These two clinical findings (i.e., restricted segment range of motion and pain elicitation on palpation over the relevant joint) are the two most reliable clinical indicators of spinal dysfunction [43,44,45,46].

For the lumbar spine, inter-segmental range of motion has also been shown to have acceptable reliability, particularly for the lower lumbar segments [47]. Although it is recognized that clinical tests of sacroiliac joint function have questionable reliability [48,49], these tests are still widely used clinically, and Flynn et al. [50] have adopted them as one of the criteria for a clinical prediction rule of whether a patient is likely to benefit from sacroiliac manipulation. For the purpose of this study lumbopelvic dysfunction was defined as the presence of both restricted intersegmental range of motion and tenderness to palpation of at least one lumbopelvic spinal joint segment.

To assess the function of the lumbar segments, the examining chiropractor palpated the movement of individual lumbar segments while the participant’s spine is laterally flexed to the right and left. Where the movement felt restricted, the examiner applied gentle pressure to the joint and surrounding soft tissues, while watching for signs of discomfort from the subject. The examiner also asked the subject if the pressure to the joint elicited pain and/or tenderness. To assess the function of the sacroiliac joints, subjects were asked to walk up and down on the spot with their knees flexed to assess the movement of each ilium relative to the sacrum while the assessor held their thumbs on the inferior margin of either the right or the left posterior iliac spines and the adjacent aspect of the sacrum. When the posterior superior iliac spine (PSIS) and the sacrum moves together, the joint was considered to be restricted in this plane. Subjects were also being asked to bend sideways while the examiner’s thumbs contacted the right and left PSISs. Subjects where the sacrum does not shift toward the contralateral side will be considered to be restricted in the lateral flexion plane. When apparent movement dysfunction was identified on one side, the clinician then palpated over sacroiliac joints and asked the participant if the palpation elicited tenderness over the joint. These two clinical findings (i.e., restricted segment range of motion and pain elicitation on palpation over the relevant joint) are the two most reliable clinical indicators of spinal dysfunction [43,44,45,46].

All spinal manipulations carried out in this study were high-velocity, low-amplitude thrusts to the spine held in lateral flexion, with slight rotation and slight extension. This is a standard manipulative technique used by chiropractors. The mechanical properties of this type of CNS perturbation have been investigated, and, although the actual force applied to the subject’s spine depends on the therapist, the patient, and the spinal location of treatment, the general shape of the force-time history of spinal manipulation is very consistent [51] and the duration of the thrust is always less than 200 milliseconds [52]. The high-velocity type of manipulation was chosen specifically because previous research [53] has shown that reflex EMG activation observed after manipulation only occurred after high-velocity, low-amplitude manipulations, (as compared with lower-velocity mobilizations). This manipulative technique has also been previously used in studies that have investigated neurophysiological effects of spinal manipulation [7,8,9,12]. 

#### 2.6.2. Control Intervention

The control intervention in Experiment 1 consisted of a passive movement of the subject’s head that was carried out by the same chiropractor who had pre-checked the subjects for spinal dysfunction and who performed the spinal manipulations for the spinal manipulation experiment. The passive head movement control intervention involved the subject’s head being passively laterally flexed, and slightly extended and rotated to a position that the chiropractor would normally manipulate that person’s cervical spine and then returning the subject’s head back to neutral position. This was repeated to both the left and the right. However, the experimenter was particularly careful not to put pressure on any individual cervical segment. This was done because previous studies have shown that loading a joint, as is done prior to spinal manipulation, has been shown to alter paraspinal proprioceptive firing in anesthetized cats [54] and was therefore carefully avoided by ending the movement before end range of motion when passively moving the subjects’ heads. No spinal manipulation was performed during any passive head movement experiment. The passive head movement experiment was the control intervention and was not intended to act as a sham manipulation but as a physiological control for possible changes occurring because of the cutaneous, muscular, or vestibular input that would occur with the type of passive head movement involved in preparing a subject/patient for a cervical manipulation. It also acted as a control for the effects of the magnetic and/or electrical stimulation necessary to collect the dependent measures of the study.

### 2.7. Statistical Analysis

A three-way repeated measures ANOVA with factors intervention (cervical spinal manipulation and control), “time” (pre, and post0), and “stimulus intensity” (90%, 100%, 110%, 120% and 130% RTh) was used to investigate the effects of the interventions on the changes in the MEP amplitude on upper limb muscle (APB). A two-way ANOVA with factors “time” (pre and post), and “stimulus intensity” (90%, 100%, 110%, 120% and 130% RTh) was used to investigate changes in MEP size prior to and following a full spinal manipulation on lower limb muscle (TA). One-way ANOVA were used to find the changes in M-wave and F-wave parameters (amplitude and persistence) with “intervention” (spinal manipulation vs. control) as factor. In addition, for MRCP morphological parameters (amplitudes and latencies), one-way ANOVA was used. Paired *t*-tests were used to assess changes in the parameters of the input–output curve (MEPmax, S_50_, the slope and the *r*^2^ value). For all experiments, statistical significance was set to *p* < 0.05.

## 3. Results

### 3.1. Upper Limb TMS

**Figure 1:**
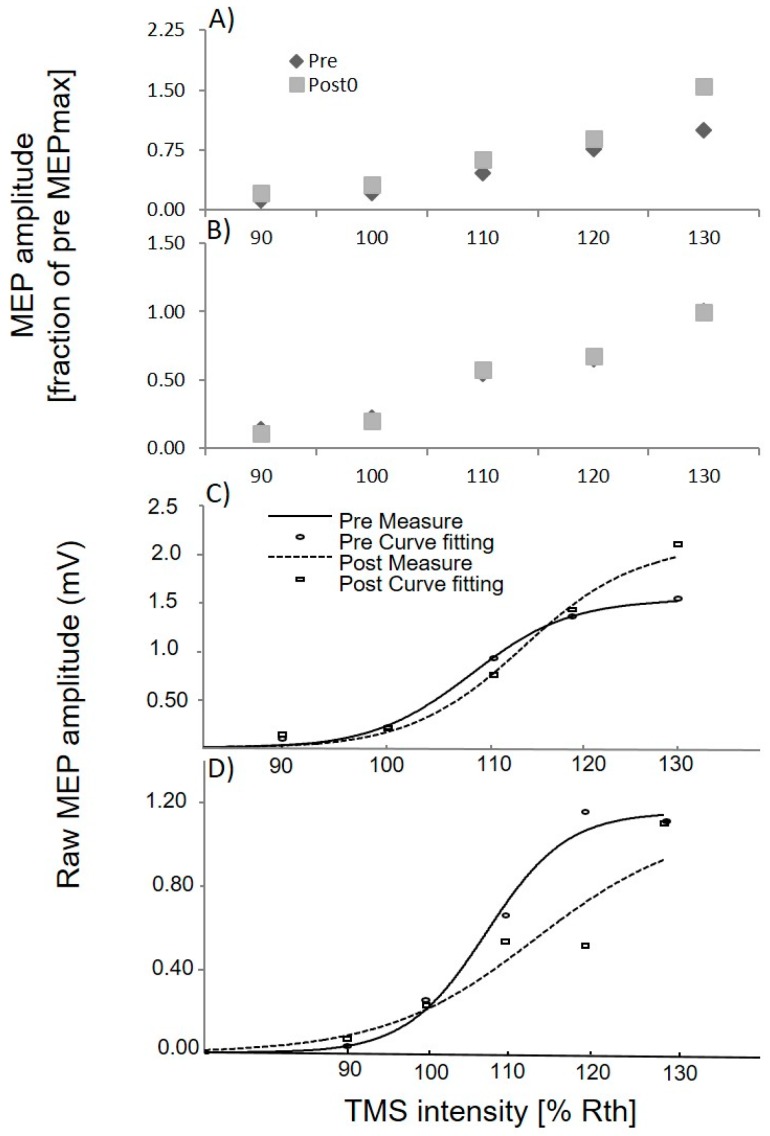
Motor evoked potential (MEP) recruitment curve for the upper limb muscle (APB) across all subjects, (**A**) prior to (pre), and following (post0) the cervical manipulation intervention. (**B**) Control Passive head movement group. Raw and fitted MEP recruitment curve for APB: (**C**) prior to, and following the cervical manipulation intervention for *n* = 1; and (**D**) prior to, and following the PHM for *n* = 1.

The APB MEP was elicited at five intensities of TMS, ranging from 90% to 130% of RTh. A Boltzmann fit was applied to the extracted raw peak to peak APB MEP’s curve, as shown in Figure 1C,D for cervical spinal manipulation and PHM respectively in a subject. On average APB MEPmax was increased by 54.51% ± 93.13% and 11.24% ± 69.95% of pre measures of MEP in cervical manipulation and PHM sessions respectively. As the MEP values varies widely across individuals so we have normalized the peak to peak APB MEPmax for each subject and all stimulation intensities to the maximum MEP recorded in the pre-intervention (MEPmax). Figure 1A shows the averaged APB MEP’s size prior to (pre) and following (post0) the cervical manipulation intervention and in PHM session (Figure 1B) in a total of 12 subjects. A three-way ANOVA with factors intervention (Cervical manipulation and PHM), “time” (pre and post0) and “stimulus intensity” (90%, 100%, 110%, 120% and 130% RTh) revealed significant three way (Intervention × time × intensities) interaction (F(4, 44) = 3.8; *p* = 0.01). Afterwards, to find out the effect of each intervention (Cervical manipulation and PHM), a two-way ANOVA was conducted individually with time’ (pre and post0) and “stimulus intensity” (90%, 100%, 110%, 120% and 130% RTh) as factors. This two-way ANOVA revealed significant difference of factor time (F(1, 11) = 5.54; *p* = 0.03) for Cervical spinal manipulation data but non-significant difference for factor time (F(1, 11) = 0.01; *p* = 0.98) in PHM data. Post hoc test revealed significantly greater APB MEPmax from pre to post0 (*p* = 0.03) in cervical spinal manipulation data and non-significant change in APB MEPmax in PHM data. Across all subjects and all sessions, the Boltzmann fit accounted for more than 80% of the total variance in the data (*r*^2^ ≥ 0.80). The S_50_ and the slope remained unchanged for both cervical manipulation and PHM data (*p* > 0.05).

### 3.2. Upper Limb F and M Waves

There were no significant changes to the APB F wave parameters measured (F wave amplitude and persistence) or M wave amplitudes. 

### 3.3. Lower Limb TMS

**Figure 2:**
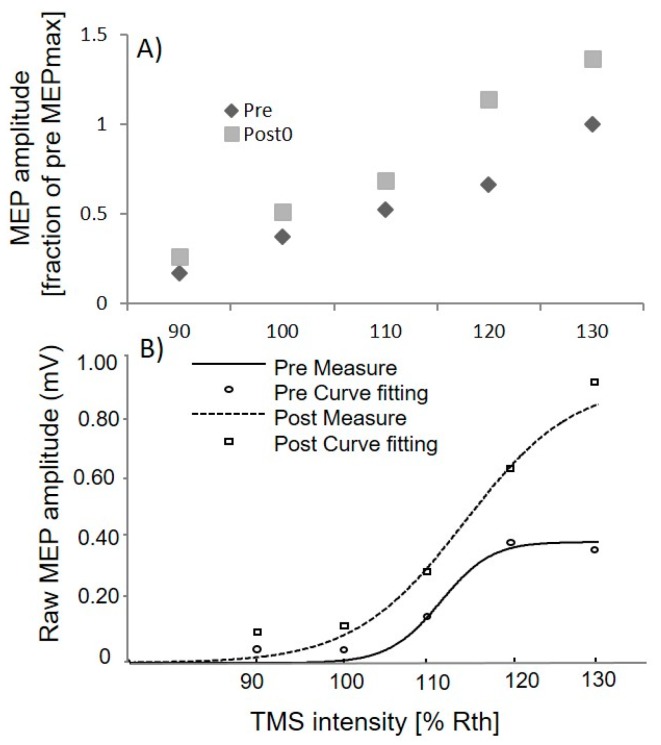
Motor evoked potential (MEP) recruitment curve prior to, and following the full spinal manipulation intervention for lower limb muscle (TA): (**A**) input–output properties of the TA MEP; and (**B**) raw and fitted TA MEP recruitment curve for *n* = 1.

The effectiveness of the full spinal manipulation on lower limb muscle (TA) was also quantified by assessing changes in the input–output curves of the TA MEP prior to (pre) and following (post0) after full spinal manipulation intervention in total of 14 subjects. A Boltzmann fit was applied to the extracted raw peak to peak TA MEP’s curve, as shown in Figure 2B for a subject. On average TA MEPmax in post measure improved by 44.56% ± 69.56% of pre measures of MEP. Figure 2A shows the averaged TA MEP size prior to (pre) and following (post0) the intervention in total of 14 subjects. As the MEP values varies widely across individuals so we have to normalized the peak to peak TA MEPmax for each subject and all stimulation intensities to the maximum TA MEP recorded in the pre-intervention (TA MEPmax). A two-way ANOVA with factors “time” (pre, post0), and “stimulus intensity” (90%, 100%, 110%, 120% and 130% RTh) revealed the significant difference for time (F(1, 13) = 6.07; *p* = 0.02). Post hoc analysis revealed significantly greater TA MEPmax from pre to post0 (*p* = 0.01). Across all subjects the Boltzmann fit accounted for more than 80% of the total variance in the data (*r*^2^ ≥ 0.80). The S_50_ and the slope value remained unchanged across intervention (*p* > 0.05).

### 3.4. Lower Limb MRCP Morphology

One-way ANOVA was used to see the changes in amplitudes and latencies of morphological parameters (EBP, LBP, PN and rebound rate of RAP). Prior to any other statistical calculation on morphological parameters one-way ANOVA was performed to see the change in force level for performed ballistic dorsiflextion of TA in pre and post intervention (full spinal manipulation) session to make sure subject were exerting same level of force in pre and post intervention session, which revealed no statistical difference (F(1, 11) = 1.07; *p* = 0.32). Furthermore, analysis of rebound rate of AP which changes with different force level [19] also revealed non-significant difference (F(1, 11) = 0.001; *p* = 0.98) from pre to post intervention session. No observed changes for these parameters support the argument that the subjects performed the dorsiflexion similarly before and after the SM intervention.

**Figure 3:**
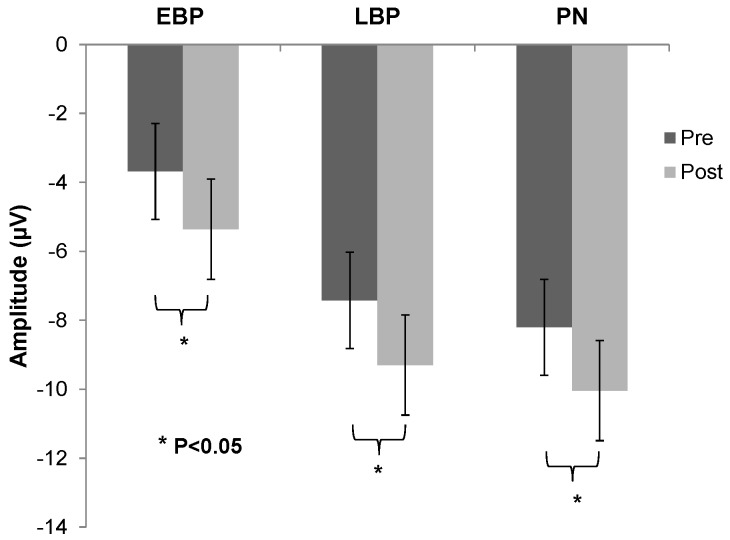
Group mean (±SE) of the early bereitschafts-potential (EBP), late bereitschafts potential (LBP) and Peak negativity (PN) amplitudes in the pre (black bars) versus post-spinal manipulation session (grey bars).

All parameters of latencies were non-significant (*p* > 0.05) prior to and after the intervention session. However, significant difference was found for amplitude of EBP (F(1, 11) = 7.32; *p* = 0.02), LBP (F(1, 11) = 6.96; *p* = 0.02) and also for PN (F(1, 11) = 6.03; *p* = 0.03) as shown in Figure 3, average across all 12 subjects.

## 4. Discussion

The results of this study show that spinal manipulation leads to short term changes in cortical excitability, as measured by a significantly larger MEPmax for TMS induced input output curves for both an upper and lower limb muscle, and with larger amplitudes of MRCP component post manipulation. No changes in spinal measures (i.e., F-wave amplitudes or persistence) were observed, and no changes were shown following the control condition.

### 4.1. Neuroplastic Changes

The current study found a significant increase in the MEPmax of the input–output curve following spinal manipulation with the data fitted to the Boltzman equation. Previous research exploring the possible neural mechanisms which underlie the shape and parameters of the input–output relation [55] have suggested that the MEPmax, also known as the plateau of the sigmoidal shaped recruitment curve most likely reflects the balance between excitatory and inhibitory components of the corticospinal volley. The finding from the current study therefore suggests that spinal manipulation alters the balance between excitatory and inhibitory components of the corticospinal volley. This is in accordance with an earlier TMS study that found muscle specific changes in intracortical inhibitory and excitatory pathways following spinal manipulation [9]. In this experiment, short-interval intracortical inhibition (SICI) and short-interval intracortical excitation (SICF) along with motor evoked potentials (MEP) and cortical silent periods (CSP) were recorded before and after either spinal manipulation of a control intervention. Muscle specific changes in SICI, SICF and CSP duration were observed following spinal manipulation only. Taken together, these findings and the results of the current experiment suggest spinal manipulation alters the balance of intracortical inhibitory and excitatory output to muscles. The previous study [9] did not find changes in MEPs for ABP at stimulus intensities of 150% active threshold. In the current study. We only recorded MEPs during rest and increasing intensities. As can be seen in Figure 1A for the upper limb (APB), only the highest intensities revealed changes in MEP amplitudes post manipulation, and this may be why no changes in MEP amplitudes where seen in the previous study, as only one intensity was assessed [9]. If using single TMS stimuli, according to the International Federation of Clinical Neurophysiology (IFCN) 2012 practical guidelines, the optimal stimulus intensity should correspond to the transition from the rising slope to the flat portion of the sigmoid stimulus–response curve [56]. The IFCN practical guidelines furthermore recommend that this optimal intensity corresponds approximately to 140% RMT or 170% active motor threshold [56]. As the previous study from 2008 [9] did not find changes in MEPs for APB at a stimulus intensities of 150% active threshold, this stimulus intensity is likely to have been lower down the stimulus response curve compared to the 170% active motor threshold that is known to generally reflect the transition from the rising slope to the flat portion. In the current study our highest stimulus intensity was 130% rest threshold, which is closer to the recommended 140% [56] reflecting this same transition point when stimulating at rest. As a flattening of the stimulus response curves can clearly be seen in the pre-intervention data in Figure 1C,D and Figure 2B, this 130% of resting threshold stimulation was clearly at that level of the response curve where it was flattening. What can also clearly be seen from Figure 1 representing the results in the upper limb is that at lower stimulus intensities than 130% rest threshold the curves overlap. Thus only at the level where the stimulus response curve flattens do we see a change in the MEP amplitude. Assuming that the previous study [9] that stimulated at 150% active threshold was producing MEPs at a level on the slope of the response curve, the findings of the current study and this previous study and congruent with each other. 

### 4.2. Cortical vs. Spinal Changes

The self-paced EBP component of the MRCP is a slow negativity beginning up to 2 s prior to movement onset. It is known to reflect motor preparatory activity, mainly from the supplementary motor area (SMA) [28,57,58,59,60,61,62]. In the current study, spinal manipulation lead to an increase in the amplitude of the EBP, suggesting a change in motor preparatory activity occurring primarily in the SMA. Multiple previous studies have shown changes that also suggest spinal manipulation alters early sensorimotor integration [7,10,11,22]. Several studies have shown changes in the amplitude of the P22-N30 somatosensory evoked potential complex [7,10,11,42]. The P22-N30 is a SEP peak known to have multiple generators [16,17,18,19,20]. Numerous authors have demonstrated that this SEP peak complex is generated by activity in a complex loop linking the Basal Ganglia, pre-motor and primary motor and somatosensory cortices [43,44,45,46,47] and is therefore thought to reflect early sensorimotor integration [21]. 

The LBP component of the MRCP is a negative deflection that begins approximately 500 ms prior to movement onset and peaks just after movement onset. This component of the MRCP is known to be primarily generated by the contralateral primary motor cortex (M1) [61,63,64,65,66,67,68]. The RAP, is a positive deflection occurring after movement onset and reflects primary somatosensory cortical (S1) activity [61,69,70,71]. The change in the MRCP morphology found in the current study corroborates previous studies that have also shown that spinal manipulation leads to changes lasting at least from 10 to 30 min on average in motor control processing at the cortical level [7,24,72]. One previous study demonstrated improvement in a motor learning task performance which occurred concurrently with a decrease in cerebellar-M1 inhibition when spinal manipulation was performed with the motor sequence learning task [72]. Although the control group (that did not receive spinal manipulation) also showed a significant improvement in task performance there were no changes observed in cerebellar-M1 inhibition. This suggests that spinal manipulation performed alongside motor learning leads to specific changes in cerebellar-M1 processing. The current study’s LBP results, known to reflect M1 processing, may therefore also be reflective of similar motor preparatory cerebellar-M1 processing changes that occur with spinal manipulation. Another study [24] has suggested that the large changes in maximum voluntary contraction (MVC) forces observed following spinal manipulation were most likely due to increased descending cortical drive because the observed H-reflex changes could not account for the large MVC changes. The current study supports these findings.

Some authors have argued that as the MRCP is time-locked with the preparation of a movement, and thus another potential neural generator should be considered. For example, sub-cortical structures that are also known to be important for motor execution, such as the basal ganglia [73] and posterior thalamus [74], have been shown to influence the morphology of the MRCP. Although such sub-cortical structures are known to be important for motor control, there is limited evidence that spinal manipulation leads to changes in sub-cortical structures. Some evidence exists for small significant changes in the H-reflex in the lower limb [24,75] However, the small changes in spinal excitability could not account for the large motor output changes observed in Niazi et al. [24].

### 4.3. Limitation

The spinal cord excitability measure used in this study, F waves, only measures a small portion of the motor unit pool tested, by anti dromically activating some of the alpha-motor neurons. It is therefore not a comprehensive measure of spinal cord excitability. In a previous study, our group has conducted an H-reflex study and did find a small but significant shift of the H-reflex threshold after spinal manipulation that was not evident following a control intervention [24]. Spinal cord excitability changes can therefore not be ruled out in this study.

It should also be noted that this current study findings cannot in any way be generalized to clinical populations. The current study only assessed subclinical spinal pain participants; in other words, relatively healthy asymptomatic subjects that occasionally suffer from intermittent pain, tension and/or ache. Future research would need to explore whether similar changes do also occur in clinical populations that have lost tonus of their muscles and/or are recovering from muscle degrading dysfunctions such as stroke or orthopaedic operations.

### 4.4. Functional Implication

This study demonstrates significant increases in corticopsinal excitability to both an upper and a lower limb muscle. This has been observed with significantly larger TMS-induced stimulus response (SR) curve MEPmax for the upper limb and an entire shift of the TMS induced SR curve to the left for the lower limb. The study results also indicate that at least in part this increased excitability occurs at the cortical level due to the increased amplitude of the MRCPs following spinal manipulation. These findings are consistent with previous findings that have suggested increases in strength following spinal manipulation were due to descending cortical drive and could not be explained by changes at the level of the spinal cord [24]. Spinal manipulation may therefore be indicated for the patients who have lost tonus of their muscle and or are recovering from muscle degrading dysfunctions such as stroke or orthopaedic operations. These results may also be of interest to sports performers. We suggest these findings should be followed up in the relevant populations.

## 5. Conclusions

The result presented are consistent with previous findings that have suggested increases in strength following spinal manipulation were due to descending cortical drive and could not be explained by changes at the level of the spinal cord. Spinal manipulation may therefore be indicated for the patients who have lost tonus of their muscle and/or are recovering from muscle degrading dysfunctions such as stroke or orthopaedic operations and/or may also be of interest to sports performers. These findings should be followed up in the relevant populations.

## References

[1] Haavik H., Murphy B. (2012). The role of spinal manipulation in addressing disordered sensorimotor integration and altered motor control. J. Electromyogr. Kinesiol..

[2] Bronfort G., Evans R., Anderson A.V., Svendsen K.H., Bracha Y., Grimm R.H. (2012). Spinal manipulation, medication, or home exercise with advice for acute and subacute neck pain: A randomized trial. Ann. Intern. Med..

[3] Bronfort G., Haas M., Evans R.L., Bouter L.M. (2004). Efficacy of spinal manipulation and mobilization for low back pain and neck pain: A systematic review and best evidence synthesis. Spine J..

[4] Kuczynski J.J., Schwieterman B., Columber K., Knupp D., Shaub L., Cook C.E. (2012). Effectiveness of physical therapist administered spinal manipulation for the treatment of low back pain: A systematic review of the literature. Int. J. Sports Phys. Ther..

[5] Gross A., Miller J., D’Sylva J., Burnie S.J., Goldsmith C.H., Graham N., Haines T., Bronfort G., Hoving J.L. (2010). Manipulation or mobilisation for neck pain: A cochrane review. Man. Ther..

[6] Marshall P., Murphy B. (2006). The effect of sacroiliac joint manipulation on feed-forward activation times of the deep abdominal musculature. J. Manip. Physiol. Ther..

[7] Haavik-Taylor H., Murphy B. (2007). Cervical spine manipulation alters sensorimotor integration: A somatosensory evoked potential study. Clin. Neurophysiol..

[8] Haavik-Taylor H., Murphy B. (2007). Transient modulation of intracortical inhibition following spinal manipulation. Chiropr. J. Aust..

[9] Taylor H.H., Murphy B. (2008). Altered sensorimotor integration with cervical spine manipulation. J. Manip. Physiol. Ther..

[10] Taylor H.H., Murphy B. (2010). Altered central integration of dual somatosensory input after cervical spine manipulation. J. Manip. Physiol. Ther..

[11] Haavik Taylor H., Murphy B. (2010). The Effects of spinal manipulation on central integration of dual somatosensory input observed after motor training: A crossover study. J. Manip. Physiol. Ther..

[12] Haavik H., Murphy B. (2011). Subclinical neck pain and the effects of cervical manipulation on elbow joint position sense. J. Manip. Physiol. Ther..

[13] Allison T., McCarthy G., Wood C.C., Williamson P.D., Spencer D.D. (1989). Human Cortical potentials evoked by stimulation of the median nerve. II. Cytoarchitectonic areas generating long-latency activity. J. Neurophysiol..

[14] Allison T., McCarthy G., Wood C.C., Darcey T.M., Spencer D.D., Williamson P.D., Spencer D.D. (1989). Human cortical potentials evoked by stimulation of the median nerve. II. Cytoarchitectonic areas generating short-latency activity. J. Neurophysiol..

[15] Allison T., Wood C.C., McCarthy G., Spencer D.D. (1991). Cortical somatosensory evoked potentials. II. Effects of excision of somatosensory or motor cortex in humans and monkeys. J. Neurophysiol..

[16] Kanovsky P., Bares M., Rektor I. (2003). The selective gating of the N30 cortical component of the somatosensory evoked potentials of median nerve is different in the mesial and dorsolateral frontal cortex: Evidence from intracerebral recordings. Clin. Neurophysiol..

[17] Mauguiere F., Desmedt J.E., Courjon J. (1983). Astereognosis and dissociated loss of frontal or parietal components of somatosensory evoked potentials in hemispheric lesions. detailed correlations with clinical signs and computerized tomographic scanning. Brain.

[18] Rossini P.M., Gigli G.L., Marciani M.G., Zarola F., Caramia M. (1987). Non-invasive evaluation of input-output characteristics of sensorimotor cerebral areas in healthy humans. Electroencephalogr. Clin. Neurophysiol..

[19] Rossini P.M., Babiloni F., Bernardi G., Cecchi L., Johnson P.B., Malentacca A., Stanzione P., Urbano A. (1989). Abnormalities of Short-latency somatosensory evoked potentials in parkinsonian patients. Electroencephalogr. Clin. Neurophysiol..

[20] Waberski T.D., Buchner H., Perkuhn M., Gobbele R., Wagner M., Kucker W., Silny J. (1999). N30 and the effect of explorative finger movements: A model of the contribution of the motor cortex to early somatosensory potentials. Clin. Neurophysiol..

[21] Rossi S., della Volpe R., Ginanneschi F., Ulivelli M., Bartalini S., Spidalieri R., Rossi A. (2003). Early somatosensory processing during tonic muscle pain in humans: Relation to loss of proprioception and motor ‘defensive’ strategies. Clin. Neurophysiol..

[22] Lelic D., Niazi I.K., Holt K., Jochumsen M., Dremstrup K., Yielder P., Murphy B., Drewes A.M., Haavik H. (2016). Manipulation of dysfunctional spinal joints affects sensorimotor integration in the prefrontal cortex: A brain source localization study. Neural Plast..

[23] Holt K.R., Haavik H., Lee A.C.L., Murphy B., Elley C.R. (2016). Effectiveness of chiropractic care to improve sensorimotor function associated with falls risk in older people: A randomized controlled trial. J. Manip. Physiol. Ther..

[24] Niazi I.K., Türker K.S., Flavel S., Kinget M., Duehr J., Haavik H. (2015). Changes in H-reflex and V-waves following spinal manipulation. Exp. Brain Res..

[25] Schlaug G., Renga V., Nair D. (2008). Transcranial direct current stimulation in stroke recovery. Arch. Neurol..

[26] Mansur C.G., Fregni F., Boggio P.S., Riberto M., Gallucci-Neto J., Santos C.M., Wagner T., Rigonatti S.P., Marcolin M.A., Pascual-Leone A. (2005). A Sham stimulation-controlled trial of rTMS of the unaffected hemisphere in stroke patients. Neurology.

[27] Hummel F., Celnik P., Giraux P., Floel A., Wu W.-H., Gerloff C., Cohen L.G. (2005). Effects of non-invasive cortical stimulation on skilled motor function in chronic stroke. Brain.

[28] Hallett M., Jahanshahi M. (2003). The Bereitschaftspotential: Movement-Related Cortical Potentials.

[29] Rossi S., Hallett M., Rossini P.M., Pascual-Leone A. (2009). Safety of TMS consensus group. safety, ethical considerations, and application guidelines for the use of transcranial magnetic stimulation in clinical practice and research. Clin. Neurophysiol..

[30] Lee H., Nicholson L.L., Adams R.D. (2004). Cervical range of motion associations with subclinical neck pain. Spine.

[31] Lee H., Nicholson L.L., Adams R.D., Bae S.-S. (2005). Proprioception and rotation range sensitization associated with subclinical neck pain. Spine.

[32] Lee H.-Y., Wang J.-D., Yao G., Wang S.-F. (2008). Association between cervicocephalic kinesthetic sensibility and frequency of subclinical neck pain. Man. Ther..

[33] Carroll T.J., Riek S., Carson R.G. (2001). Reliability of the input-output properties of the cortico-spinal pathway obtained from transcranial magnetic and electrical stimulation. J. Neurosci. Methods.

[34] Malcolm M.P., Triggs W.J., Light K.E., Shechtman O., Khandekar G., Gonzalez Rothi L.J. (2006). Reliability of motor cortex transcranial magnetic stimulation in four muscle representations. Clin. Neurophysiol..

[35] Kamen G. (2004). Reliability of motor-evoked potentials during resting and active contraction conditions. Med. Sci. Sports Exerc..

[36] Fisher M.A. (1992). AAEM Minimonograph #13: H reflexes and F waves: Physiology and clinical indications. Muscle Nerve.

[37] Panayiotopoulos C.P., Chroni E. (1996). F-Waves in clinical neurophysiology: A review, methodological issues and overall value in peripheral neuropathies. Electroencephalogr. Clin. Neurophysiol..

[38] Taniguchi M.H., Hayes J., Rodriguez A.A. (1993). Reliability determination of F mean response latency. Arch. Phys. Med. Rehabil..

[39] Slobounov S.M., Ray W.J. (1998). Movement-related potentials with reference to isometric force output in discrete and repetitive tasks. Exp. Brain Res..

[40] Do Nascimento O.F., Nielsen K.D., Voigt M. (2005). Relationship between plantar-flexor torque generation and the magnitude of the movement-related potentials. Exp. Brain Res..

[41] Hestbaek L., Leboeuf-Yde C. (2000). Are Chiropractic Tests for the lumbo-pelvic spine reliable and valid? A systematic critical literature review. J. Manip. Physiol. Ther..

[42] Fryer G., Morris T., Gibbons P. (2004). Paraspinal muscles and intervertebral dysfunction: Part one. J. Manip. Physiol. Ther..

[43] Jull G., Bogduk N., Marsland A. (1988). The Accuracy of manual diagnosis for cervical zygapophysial joint pain syndromes. Med. J. Aust..

[44] Hubka M.J., Phelan S.P. (1994). Interexaminer reliability of palpation for cervical spine tenderness. J. Manip. Physiol. Ther..

[45] Rheault W., Albright B., Beyers C., Franta M., Johnson A., Skowronek M., Dougherty J. (1992). Intertester reliability of the cervical range of motion device. J. Orthop. Sports Phys. Ther..

[46] Youdas J.W., Garrett T.R., Suman V.J., Bogard C.L., Hallman H.O., Carey J.R. (1992). Normal range of motion of the cervical spine: An initial goniometric study. Phys. Ther..

[47] Strender L.E., Sjoblom A., Sundell K., Ludwig R., Taube A. (1997). Interexaminer reliability in physical examination of patients with low back pain. Spine.

[48] Herzog W., Read L.J., Conway P.J., Shaw L.D., McEwen M.C. (1989). Reliability of motion palpation procedures to detect sacroiliac joint fixations. J. Manip. Physiol. Ther..

[49] Potter N.A., Rothstein J.M. (1985). Intertester reliability for selected clinical tests of the sacroiliac joint. Phys. Ther..

[50] Flynn T., Fritz J., Whitman J., Wainner R., Magel J., Rendeiro D., Butler B., Garber M., Allison S. (2002). A clinical prediction rule for classifying patients with low back pain who demonstrate short-term improvement with spinal manipulation. Spine.

[51] Hessell B.W., Herzog W., Conway P.J., McEwen M.C. (1990). Experimental measurement of the force exerted during spinal manipulation using the thompson technique. J. Manip. Physiol. Ther..

[52] Herzog W., Lawrence D. (1996). Mechanical, Physiologic, and Neuromuscular Considerations of Chiropractic Treatment.

[53] Herzog W., Conway P.J., Zhang Y.T., Gal J., Guimaraes A.C. (1995). Reflex responses associated with manipulative treatments on the thoracic spine: A pilot study. J. Manip. Physiol. Ther..

[54] Pickar J.G., Wheeler J.D. (2001). Response of Muscle proprioceptors to spinal manipulative-like loads in the anesthetized cat. J. Manip. Physiol. Ther..

[55] Devanne H., Lavoie B.A., Capaday C. (1997). Input-output properties and gain changes in the human corticospinal pathway. Exp. Brain Res..

[56] Groppa S., Oliviero A., Eisen A., Quartarone A., Cohen L.G., Mall V., Kaelin-Lang A., Mima T., Rossi S., Thickbroom G.W. (2012). A practical guide to diagnostic transcranial magnetic stimulation: Report of an IFCN committee. Clin. Neurophysiol..

[57] Deecke L., Kornhuber H.H. (1978). An electrical sign of participation of the mesial ‘supplementary’ motor cortex in human voluntary finger movement. Brain Res..

[58] Deecke L. (1987). Bereitschaftspotential as an indicator of movement preparation in supplementary motor area and motor cortex. Ciba Found. Symp..

[59] Deecke L., Lang W. (1996). Generation of movement-related potentials and fields in the supplementary sensorimotor area and the primary motor area. Adv. Neurol..

[60] Praamstra P., Stegeman D.F., Horstink M.W., Cools A.R. (1996). Dipole source analysis suggests selective modulation of the supplementary motor area contribution to the readiness potential. Electroencephalogr. Clin. Neurophysiol..

[61] Shibasaki H., Hallett M. (2006). What is the bereitschaftspotential?. Clin. Neurophysiol..

[62] Cui R., MacKinnon C.D. (2009). The effect of temporal accuracy constraints on movement-related potentials. Exp. Brain Res..

[63] Deecke L., Scheid P., Kornhuber H.H. (1969). Distribution of readiness potential, pre-motion positivity, and motor potential of the human cerebral cortex preceding voluntary finger movements. Exp. Brain Res..

[64] Deecke L., Grozinger B., Kornhuber H.H. (1976). Voluntary finger movement in man: cerebral potentials and theory. Biol. Cybern..

[65] Kristeva R., Keller E., Deecke L., Kornhuber H.H. (1979). Cerebral potentials preceding unilateral and simultaneous bilateral finger movements. Electroencephalogr. Clin. Neurophysiol..

[66] Kutas M., Donchin E. (1980). Preparation to respond as manifested by movement-related brain potentials. Brain Res..

[67] Shibasaki H., Barrett G., Halliday E., Halliday A.M. (1980). Components of the movement-related cortical potential and their scalp topography. Electroencephalogr. Clin. Neurophysiol..

[68] Barrett G., Shibasaki H., Neshige R. (1985). A Computer-assisted method for averaging movement-related cortical potentials with respect to EMG onset. Electroencephalogr. Clin. Neurophysiol..

[69] Kornhuber H.H., Deecke L. (1965). Changes in the brain potential in voluntary movements and passive movements in man: Readiness potential and reafferent potentials. Pflugers Arch. Gesamte Physiol. Menschen Tiere.

[70] Vaughan H.G.J., Costa L.D., Ritter W. (1968). Topography of the human motor potential. Electroencephalogr. Clin. Neurophysiol..

[71] Colebatch J.G. (2007). Bereitschaftspotential and movement-related potentials: origin, significance, and application in disorders of human movement. Mov. Disord..

[72] Daligadu J., Haavik H., Yielder P.C., Baarbe J., Murphy B. (2013). Alterations in cortical and cerebellar motor processing in subclinical neck pain patients following spinal manipulation. J. Manip. Physiol. Ther..

[73] Alexander G.E. (1994). Basal Ganglia-thalamocortical circuits: Their role in control of movements. J. Clin. Neurophysiol..

[74] Rektor I., Kanovsky P., Bares M., Louvel J., Lamarche M. (2001). Event-related potentials, CNV, readiness potential, and movement accompanying potential recorded from posterior thalamus in human subjects. A SEEG study. Neurophysiol. Clin..

[75] Murphy B.A., Dawson N.J., Slack J.R. (1995). sacroiliac joint manipulation decreases the H-reflex. Electromyogr. Clin. Neurophysiol..

